# Approaches for evaluation of novel CPP-based cargo delivery systems

**DOI:** 10.3389/fphar.2022.1056467

**Published:** 2022-10-21

**Authors:** Ly Porosk, Ülo Langel

**Affiliations:** ^1^ Laboratory of Drug Delivery, Institute of Technology, Faculty of Science and Technology, University of Tartu, Tartu, Estonia; ^2^ Department of Biochemistry and Biophysics, Stockholm University, Stockholm, Sweden

**Keywords:** cell-penetrating peptides, cargo delivery, transfection, nucleic acid therapeutics, evaluation approaches

## Abstract

Cell penetrating peptides (CPPs) can be broadly defined as relatively short synthetic, protein derived or chimeric peptides. Their most remarkable property is their ability to cross cell barriers and facilitate the translocation of cargo, such as drugs, nucleic acids, peptides, small molecules, dyes, and many others across the plasma membrane. Over the years there have been several approaches used, adapted, and developed for the evaluation of CPP efficacies as delivery systems, with the fluorophore attachment as the most widely used approach. It has become progressively evident, that the evaluation method, in order to lead to successful outcome, should concede with the specialties of the delivery. For characterization and assessment of CPP-cargo a combination of research tools of chemistry, physics, molecular biology, engineering, and other fields have been applied. In this review, we summarize the diverse, *in silico*, *in vitro* and *in vivo* approaches used for evaluation and characterization of CPP-based cargo delivery systems.

## Introduction

The advancement of omics have led to an increase of identification of new targets for addressing common diseases, such as cancers, neurodegenerative diseases, or chronic inflammation. The successful delivery of therapeutic and diagnostic cargo is required to reach new or known targets that have previously been considered hard or impossible to address. Overcoming delivery limitations is required for successful application of macromolecular drugs or for safer application of small molecule drugs. The delivery method or vector should mediate the successful delivery of the cargo, weather by increasing its stability, improving its tissue targeting, enhancing its cellular uptake, or by other means. One of the potential delivery vectors for therapeutic and diagnostic applications are the CPPs.

The CPPs are broadly defined as relatively short (∼5–40 amino acid residues (aa)) peptides, generally comprised of cationic or amphipathic membrane-interactive sequences that can cross the cell membranes (Langel and CPP, 2019). The near 2,000 known and predicted CPPs include protein transduction domains, Trojan peptides, arginine-rich peptides, bioportides, and many others (Agrawal et al., 2016). CPPs can be broadly classified into protein derived, chimeric and synthetic CPPs, although several other classifications are possible, such as based on their conformation, physico-chemical properties, or even the cargo they have delivered into the cell (Agrawal et al., 2016; Xie et al., 2020). The CPPs offer possibility to deliver a range of versatile cargo, such as fluorophores, nucleic acids, drugs, peptides, peptide nucleic acids (Singh et al., 2018; Damase et al., 2021; Yokoo et al., 2022), and enhance the uptake of cargoes with greater molecular weight compared to their own, such as proteins and nanoparticles. The nanoparticles which have been modified with CPPs include, among others, liposomes, iron oxide, and even exosomes (Chiarpotti et al., 2021; El-Gamal et al., 2022; Silva et al., 2022; Zhu et al., 2022). The versatility of CPPs and CPP-cargo associations make the evaluation of their efficacy complicated. In this work we discuss the different approaches used to evaluate CPP and CPP-cargo as cargo delivery systems.

### Cargos for CPPs

In the CPP-field the fluorophore attachment is used mostly for evaluation purposes of confirming association, cellular entrance, biodistribution *in vivo*, of the conjugated CPP. Although the attachment of a fluorophore may alter the CPP properties, internalization, cellular distribution, effects on cellular viability (Birch et al., 2017; Hedegaard et al., 2018), it is still widely used as the fluorophore can be easily detected and quantified. The fluorophore can be considered as a model cargo although the fluorescent probes, more specifically targeted fluorescent probes, have an application in fluorescence imaging-guided surgery (Liu et al., 2021).

**FIGURE 1:**
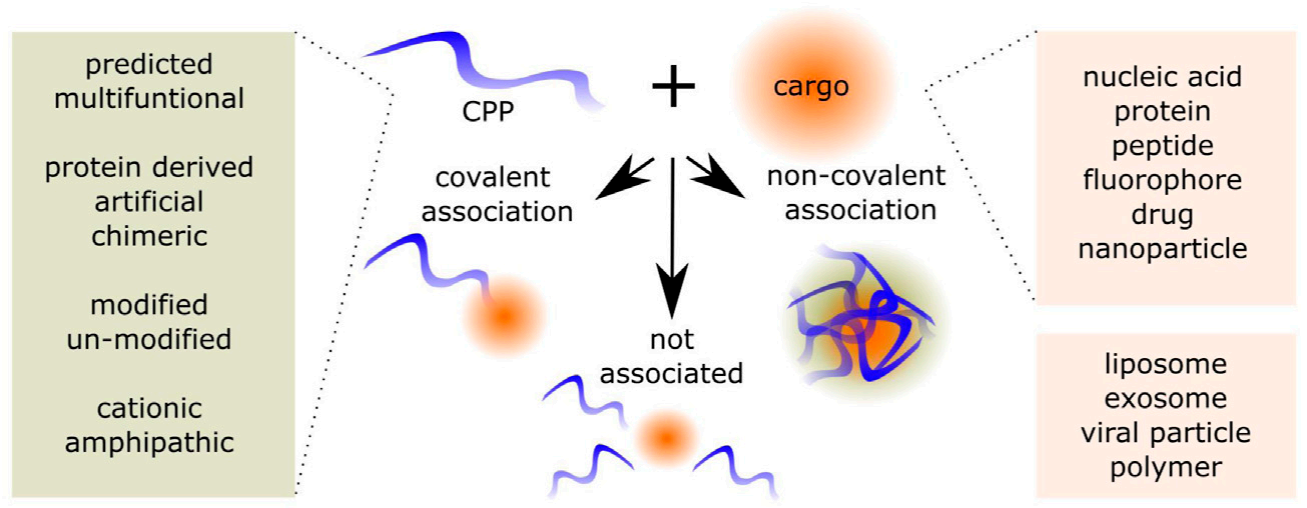
Cell-penetrating peptide (CPP) and cargo associations. The CPPs include predicted and already confirmed CPPs derived from proteins, chimeric and of artificial origin. The CPPs are a versatile family of chemical delivery vectors, where cationic, primary and secondary amphipathic, and even negatively charged peptides are represented. The CPPs may be modified to include non-proteogenic amino acid residues, addition of PEG or fatty acid residues. The cargos CPPs can be associated include a variety of moieties, additionally, CPPs can be used to enhance the internalization of NPs. The associations are the covalent association of CPP and cargo, non-covalent associations, or the CPP can be added into the mixes without it associating with the components.

Besides fluorophores, nucleic acid molecules (NA), have been widely associated with the CPP (Agrawal et al., 2016), either *via* covalent or non-covalent association, as show on Figure 1. During the recent years, there has been an increase of approved NA-based therapeutics (Kulkarni et al., 2021; Talap et al., 2021; Zhu et al., 2022). NA, which include small interfering RNA (siRNA), microRNA (miRNA), plasmid DNA (pDNA), antisense oligonucleotides (ASO), messenger RNA (mRNA), can be used for transient or long-lasting effects by inhibition, addition, editing or replacement of the target. The (unmodified) NA is susceptible to degradation by nucleases, can lead to immune activation, and is not able to enter the cells itself due to its physico-chemical properties, such as molecular weight and negative charge (Iversen et al., 2013; Kulkarni et al., 2021). Additionally, to its therapeutic applications, the NA can be used in different reporter systems. This simplifies the preliminary evaluation of CPP-NA efficacies. In addition, there is a variety of approaches to measure, label, quantify, and detect NA-s, which additionally improve the possibilities of evaluation. As mentioned above, some of the limitations of NA-based molecules are known, therefore the experiments and evaluation methods can be chosen accordingly to reflect the stability, association, or immune-activation of the CPP-NA associations.

Another class of cargo are proteins and peptides. The extracellular proteins and peptides are generally impermeable to the cell membrane, and their entrance into the cells has to be mediated by a delivery method. For example, an extracellular (therapeutic) protein requires delivery vector for cell entry (Kristensen et al., 2016). The association of proteins or peptides to the CPPs is achieved by either chemical cross-linking or cloning and expression of a protein fused to the CPP, or by non-covalent association (Gros et al., 2006; Wang et al., 2014; Kristensen et al., 2016). CPPs can enhance the uptake of both peptides, for example glucagon-like peptide-2 internalization improved with attachment of CPP R8 (Akita et al., 2021), and proteins, such as EGFP protein by Pas2r12 (Okuda et al., 2019). Besides EGFP, other reporter or model proteins with different size and properties can be used, such as beta-galactosidase.

Nanoparticles include organic nanoparticles (NP), such as micelles, liposomes, dendrimers, viral particles, and inorganic nanoparticles, such as gold or iron oxide. CPPs have been associated with the NPs by electrostatic interactions, covalent linking or even as an additive (Váňová et al., 2019; Gessner and Neundorf, 2020). Liposomes are composed of lipid bilayers with an internal water phase. Liposomes have been prepared for example using cationic lipid DOTAP, PEG, cholesterol and phosphatidylcholine and loaded with doxorubicin (DOX), and conjugated with CPP R8 to form R8PLP liposomes (Yuan et al., 2019). From the recent works there have been reports of adding CPP-conjugates to the formulations, such as R_8_-(SG)_5_-lipid grafted to PEGylated liposomes (El-Gamal et al., 2022), and also reports of enhanced uptake due to CPPs, such as transportan enhancing liposome internalization as a bystander (Li et al., 2022). Tat peptide has been used for modifying of superparamagnetic nanoparticles (Zhao et al., 2002), and tobacco mosaic virus particles (Tian et al., 2018). Additionally to these, different drugs have been added to the CPP-s to improve their efficacy or to reduce required dose limiting the side-effects of the drugs (Xie et al., 2020; Kiisholts et al., 2021).

When the cargo cannot be detected, quantified, or otherwise measured, the model cargoes belonging to the same class should be used. Nevertheless, additional functional evaluations are beneficial, with clinically relevant cargoes, such as cancer drug doxorubicin (DOX), or siRNA-s against therapeutically relevant targets, such as vascular endothelial growth factor (VEGF). There are some generally accepted methods that are often used for the initial evaluation or characterization of novel CPP-cargo associations, depending on the cargo, for example, evaluation of drug loading, the efficacy of the CPP in condensing nucleic acid cargo, or assessing the internalization of a CPP-fluorophore conjugate into cells using confocal microscopy or flow cytometry. More specific approaches are used to further characterize the association-dissociation of CPP-cargo, internalization or biodistribution. It should be kept in mind that the generalizations on CPP-cargo efficacies should be made upon the results of several complementing approaches, as every one of these have some limitations which may lead to false or biased interpretations. Often the evaluation method is dependent on the cargo, and the final goal of the delivery. Although reporter expression vectors, reporter proteins, and simplified models help to evaluate the CPP-cargo to a certain degree, clinically relevant models and achieved bioeffect in these should be evaluated.

### Assessment of CPP-cargo efficacies

The evaluation of a new CPP-cargo delivery system often begins with the characterization of the CPP and the CPP-cargo associations. After association is confirmed, internalization and trafficking of the CPP-cargo is further investigated. Additional experiments in animal models are required for preclinical evaluation if the use would include *in vivo* applications. The approaches used for the evaluation of the CPP-cargo can be broadly divided into three categories: *in silico*, *in vitro* and *in vivo*. The level at which the delivery system should be tested depends on the application. For example, the application of CPPs as plasmid delivery vectors for recombinant protein production in mammalian cell cultures was assessed on CHO and HEK293 suspension cell cultures (Porosk et al., 2022). In contrast, the evaluation of the CPP-cargo for therapeutic purposes must include preclinical assessment.

**FIGURE 2:**
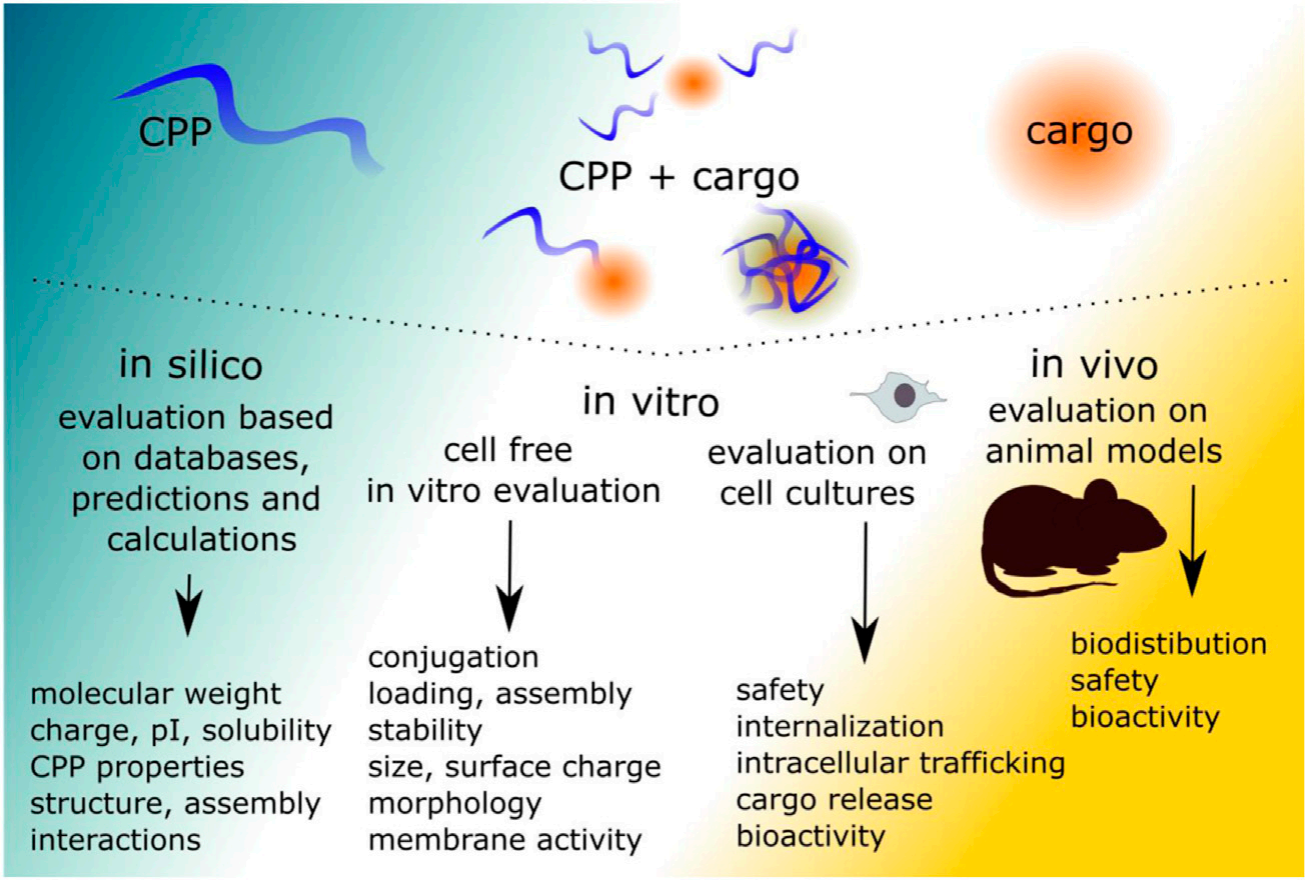
The evaluation of the CPP-cargo consists of several levels. The initial evaluation can be done by using *in silico* approaches, but as CPP-cargo systems are complicated, the evaluation should be continued to determine the aspects of the CPP-cargo. If the desired CPP-cargo characteristics have been optimized, the evaluation is continued on cell cultures. For preclinical evaluation *in vivo* models are required. The evaluation requires all these steps, as single approach may lead to false interpretations.

The assessment approaches in the following paragraphs are divided based on the general process of CPP-cargo testing, starting from the CPP design, characterization, followed by internalization, trafficking, bio-activity assessments and finally *in vivo* considerations (Figure 2). For widely used CPPs, such as tat, transportan and polyarginines, several aspects are already known based on previous works. Nevertheless, the first evaluation steps, including confirmation of linking or association are often included as cargo and attachment strategy may affect the CPP. Some methods are used to investigate several aspects of the CPP-cargo and its delivery. Many approaches used in the CPP field are well established and adapted from the fields of proteins, characterization of antimicrobial peptides and nanoparticles, drug discovery, and others. Therefore, troubleshooting guidelines are available for these regardless of their lesser use in CPP field this far.

#### *In silico* prediction and characterization of CPPs

The discovery and designing of new CPPs is a multistep process (Kardani et al., 2019; Langel and CPP, 2019; Porosk et al., 2021). Predictions, calculations and modeling may help in design and optimization of the CPPs. Often new CPP-cargo associations are created by using previously known CPPs such as TAT, PEP-1, polyarginines, penetratin, or transportan. These have been proven to be efficient by several groups, and CPP-cargo formed with these have reached to several clinical and preclinical trials.

By including modifications, such as replacing or adding new amino acid residues to the sequence, or adding cancer-targeting elements (Feni et al., 2019; Säälik et al., 2019), new CPPs with increased efficacy at specific conditions (Reissmann and Filatova, 2021; Szabó et al., 2022) can be generated. The CPP family includes peptides from ranging degrees of structural complexity and high chemical versatility. There are some common features, such as their high number of arginine or lysine residues in the sequence, length range, solubility, cationic or amphipathic nature (Derakhshankhah and Jafari, 2018), associated with CPPs, although some of them fall out of general rules associated with these peptides (de Oliveira et al., 2021). Based on these common features and databases of CPPs, such as CPPsite 2.0 (Agrawal et al., 2016), predictors for CPPs have been developed which allow to design new and unique CPPs. Additionally to CPPs databases and predictors, other peptides with specific properties, such as blood-brain barrier-penetrating peptides in B3Pdb (Kumar et al., 2021a; Kumar et al., 2021b), antimicrobial peptides in DBAASP (Pirtskhalava et al., 2021), or anticancer peptides in CancerPPD (Tyagi et al., 2015) can be found/predicted. Several groups have focused on the advancement of the prediction methods. The prediction, more importantly accurate prediction, would help to select the most promising candidates without the need to imply experiments on large libraries.

##### Prediction of CPPs

Some of CPP predictors have been reviewed in our previous work (Porosk et al., 2021). Some of CPP predictors are CPPpred (Holton et al., 2013), KELM-CPPpred (Pandey et al., 2018) MLCCP and its advancement MLCPP 2.0 (Manavalan and Patra, 2022), predictions based on aa composition, prediction from sequences including modified peptides, which contain natural and chemically modified residues Cell-MOD (Kumar et al., 2018), and predictors such as BChemRF-CPPred (de Oliveira et al., 2021) or TargetCPP (Arif et al., 2020). These predictors generally help with the design of a first generation CPP, but may also help to further modify already known CPPs to suit specific cargo and application. There are still some limitations on current prediction models as they are dependent on the quality of input data and the data used for training (Yadahalli and Verma, 2020).

##### Prediction of multifunctional peptides

In the recent years there have been progressive number of works where *in silico* approaches are combined with *in vitro* experiments, leading to the successful discovery and characterization of new CPP-based carriers and CPP-cargo moieties. Some of the examples include the design, characterization, and experimental validation of arginine-rich CPPs for plasmid delivery (Mahjoubin-Tehran et al., 2022), new CPPs predicted from SARS-CoV-2 (Henao et al., 2022), and new peptides with CPP-potential derived from corn silk (Ong et al., 2021). By using *in silico* artificial evolutionary optimization “mate-and-check” process approach and combining it with *in vitro* performance test Krause et al. achieved increased cellular uptake of new generation CPPs (Krause et al., 2018; Röckendorf et al., 2022). By integrating bioactivity into the CPP sequence, it is possible to create multifunctional peptides, where the “cargo” is the peptide. *In silico* prediction of bioactive peptides from giant African snail *Achatina fulica* mucus peptidome yielded in AMP, anti-biofilm, cytotoxic and CPP peptides (Chalongkulasak et al., 2022) by combining the results from several predictors. Machine-learning methods have been used to design short selective CPPs for targeted cells, so-called “moonlighting” short CPPs, where the reduction in size is accomplished by embedding two or more activities within a single CPP domain (Morán-Torres et al., 2021). Form 57 bioactive peptides identified from *Thalassophryne nattereri* natterin toxins by using several web servers, physiochemical properties, immunogenicity, toxicity, allergenicity analysis, and 15 potential lead compounds were selected, synthesized, and tested experimentally (De Cena et al., 2022).

Predictors allow to predict if the peptide has additional features or aid in modifying the sequences to add these features to the CPP. For this, among many others, antimicrobial peptide predictor iAMPpred, anti-biofilm predictor dPABBs, Cell-Penetrating Anticancer Peptide Predictior CpACpP, or AntiCP 2.0 can be used (Sharma et al., 2016; Meher et al., 2017; Agrawal et al., 2020; Nasiri et al., 2021). Additionally, different databases can be used as sources or for further modifications for bioactive peptides, for example for protein-protein interactions such as BioGRID, peptide-inhibitor design with PinaColada, or binding affinity estimation of peptide inhibitors with PepCrawler (Donsky and Wolfson, 2011; Zaidman and Wolfson, 2016; Oughtred et al., 2019). Additional features, such as signal sequences can also be predicted with tools such as PrediSi and Signal-IP (Almagro Armenteros et al., 2019).

##### Prediction, calculation and modeling of peptide properties

Physio-chemical parameters such as molecular weight, composition, pI, secondary structure can be calculated or predicted. Solubility of the peptide can be calculated with CamSol v2.2 or PPC ver 3.1 (Sormanni et al., 2015). Other calculators can be used for these, such as ProtScale or ProParam form Expasy, or CALCAMPI and TYPE-PEPTIDE (Gasteiger et al., 2003; Gómez et al., 2017). For example snakin peptides were selected based on their protein-binding potential Boman index and Wimley-White index, and additional parameters such as amphiphilicity index, propensity to *in vitro* aggregate (Ghanbarzadeh et al., 2022). The Boman index estimates protein-binding potential and is calculated on the basis of the cyclohexane-to-water partition coefficient of the respective amino acid side chains divided by the total number of amino acid residues within the peptide (Boman, 2003). Although generally also calculated, experimental determination of peptide octanol-water partitioning, especially in the earlier works, has been used. In the recent works it was used for characterizing peptides based on canavanine and the results indicated that CPPs based on this would not be effective (Calabretta et al., 2022). Different servers and tools for calculation and prediction are listed in Table 1.

**TABLE 1:** Online databases and tools to predict and characterize CPPs, bioactive CPPs and CPP-cargo.

Server/predictor/tool and reference	Link, short description
Peptide property calculators and predictors	
ProtScale, ProtParam (Gasteiger et al., 2003)	Compute and represent the profile produced by any amino acid scale on a selected protein (peptide)
	https://web.expasy.org/protscale/
	molecular weight, theoretical pI, amino acid composition, atomic composition, extinction coefficient, estimated half-life, instability index, aliphatic index and grand average of hydropathicity (GRAVY)
	https://web.expasy.org/protparam/
Peptide Property Calculator Ver 3.1	Theoretical calculation of peptide composition, Ip, hydrophobicity, secondary structure, transmembrane region *etc.*
	https://www.biosyn.com/peptidepropertycalculator/peptidepropertycalculator.aspx
CALCAMPI, TYPE-PEPTIDE (Gómez et al., 2017)	Molecular Weight, Net Charge, Hydrophobicity, Boman index, Aliphatic index, % of hydrophobic amino acids, Isoelectric Point and structure secondary prediction
	https://ciencias.medellin.unal.edu.co/gruposdeinvestigacion/prospeccionydisenobiomoleculas/InverPep/public/home_en
CamSol v2.2, S2Dv2 (Sormanni et al., 2015)	Prediction of protein (peptide) solubility
	http://www-vendruscolo.ch.cam.ac.uk/camsolmethod.html
	Ordered and disordered region
	https://www-cohsoftware.ch.cam.ac.uk/index.php/s2D
NetSurfP 2.0 (Klausen et al., 2019)	Predicts the surface accessibility, secondary structure, disorder, and phi/psi dihedral angles of amino acids in an amino acid sequence
	https://services.healthtech.dtu.dk/service.php?NetSurfP-2.0
TMHMM 2.0, DeepTMHMM (Möller et al., 2001; Hallgren et al., 2022)	Predictors for cellular localization
	Prediction of transmembrane helices in proteins
	https://services.healthtech.dtu.dk/service.php?TMHMM-2.0
	prediction of the membrane topology of transmembrane proteins
	https://services.healthtech.dtu.dk/service.php?DeepTMHMM
CELLPM 2.0 (Lomize et al., 2021)	Membrane interaction evaluation, a physics-based computational tool for analysis of peptide-membrane interactions and prediction of membrane permeation
	https://cellpm.org/cellpm_server
DispHred (Santos et al., 2020)	SVM-based predictor of pH-dependent folded and unfolded states
	https://ppmclab.pythonanywhere.com/DispHred
IUPred2, IUPred3 (Mészáros et al., 2018; Erdős et al., 2021)	To determine if the peptide is disordered
	https://iupred2a.elte.hu/
I-TASSER (Zheng et al., 2021)	3D structure prediction, verification, modeling
	protein structure prediction and structure-based function annotation
	https://zhanggroup.org/I-TASSER/
PrDOS (Ishida and Kinoshita, 2007)	Peptide disorder predictor
	https://prdos.hgc.jp/cgi-bin/submit.cgi
PEP-FOLD3 (Lamiable et al., 2016)	Predicts peptide structures *de novo* based on primary amino acid sequences
	https://bioserv.rpbs.univ-paris-diderot.fr/services/PEP-FOLD3
PROCHECK (Laskowski et al., 1996)	checks the stereochemical quality of a protein structure, producing a number of PostScript plots analysing its overall and residue-by-residue geometry
	https://www.ebi.ac.uk/thornton-srv/software/PROCHECK/
PinaColada (Zaidman and Wolfson, 2016)	Peptide-inhibitor prediction
	http://bioinfo3d.cs.tau.ac.il/PinaColada/
PepCrawler (Donsky and Wolfson, 2011)	Prediction of peptide-protein complexes for inhibitor design
	http://bioinfo3d.cs.tau.ac.il/PepCrawler/
Heliquest, NetWheels (Gautier et al., 2008)	Helical wheel diagram of peptides was defined by Schiffer Edmundson wheel modeling
	https://heliquest.ipmc.cnrs.fr/
	Helical wheel projection
	http://lbqp.unb.br/NetWheels/
BeStSel (Micsonai et al., 2022)	Data analysis from CD spectra measurements, secondary structure prediction
	https://bestsel.elte.hu/index.php
Antimicrobial, antiviral peptide predictors and databases	
AMPA (Torrent et al., 2012)	Predictor of antimicrobial regions from proteins. https://tcoffee.crg.eu/apps/ampa/do
DBAASP (Pirtskhalava et al., 2021)	Antimicrobial, antifungal, anticancer peptide database
	http://dbaasp.org/home
iAMPpred (Meher et al., 2017)	Prediction of antimicrobial peptides
AmpGram (Burdukiewicz et al., 2020)	Prediction and design of antimicrobial peptides
	http://biongram.biotech.uni.wroc.pl/AmpGram/
APD3 (Wang et al., 2016)	Evaluation of Boman index and protein-binding potential
	antimicrobial peptide database
	http://aps.unmc.edu/AP/
Meta-iAVP (Schaduangrat et al., 2019a)	A sequence-based meta-predictor for improving the prediction of antiviral peptides using effective feature representation
	http://codes.bio/meta-iavp
dPABBs (Sharma et al., 2016)	anti-biofilm predictor and design tool dPABBs
	http://ab-openlab.csir.res.in/abp/antibiofilm/
Anticancer	
CancerPPD (Tyagi et al., 2015)	Anticancer peptide database
	http://crdd.osdd.net/raghava/cancerppd/
AntiCP 2.0 (Agrawal et al., 2020)	Anticancer peptide prediction
	https://webs.iiitd.edu.in/raghava/anticp2/
CpACpP (Nasiri et al., 2021)	Prediction of cell-penetrating anticancer peptides
	http://cbb1.ut.ac.ir/CpACpP/Index
ACPred (Schaduangrat et al., 2019b)	A computational tool for the prediction and analysis of anticancer peptides
	http://codes.bio/acpred/
Other predictors and databases	
B3Pdb (Kumar et al., 2021a)	Database for blood-brain-barrier penetrating peptides
	https://webs.iiitd.edu.in/raghava/b3pdb/
BioGRID (Oughtred et al., 2019)	Database of protein, genetic and chemical interactions
	https://thebiogrid.org/
CPPSite 2.0 (Agrawal et al., 2016)	CPP database
	http://crdd.osdd.net/raghava/cppsite/
PrediSi (Hiller et al., 2004)	prediction of signal peptides and their cleavage positions
	http://www.predisi.de/
Signal-IP, Signal IP 5.0 (Almagro Armenteros et al., 2019)	Signal peptide and cleavage sites in gram+, gram− and eukaryotic amino acid sequences https://services.healthtech.dtu.dk/service.php?SignalP-5.0
Prediction of allergenicity, toxicity, hemolytic properties	
AllerTop, AllergenFP (Dimitrov et al., 2014)	Prediction of allergenicity
	https://www.ddg-pharmfac.net/AllerTOP/
	http://ddg-pharmfac.net/AllergenFP/
ToxinPred (Gupta et al., 2013; Gupta et al., 2015)	Analysis of toxicity and allergenicity
	https://webs.iiitd.edu.in/raghava/toxinpred/algo.php
HemoPI (Chaudhary et al., 2016)	SVM-based prediction of hemolytic activity
	https://webs.iiitd.edu.in/raghava/hemopi/design.php
IEDB, IEDB Immunogenicity Predictor (Calis et al., 2013; Chaudhary et al., 2016)	The Immune Epitope Database, prediction and analysis of immune epitopes
	https://www.iedb.org/
	http://tools.iedb.org/immunogenicity/

##### Calculation and modeling of CPP-cargo properties

Although many predictors do not consider the cargo, the parameters from the CPP calculations may be still useful for evaluation purposes. The properties of the CPP-cargo should be investigated in addition to free CPP, and the calculations, predictions based on only the CPP cannot be interpreted directly into processes when cargo is associated. The interactions between the CPP and cargo can be affected by the secondary structure of the peptide. For secondary structure, additionally to previously mentioned calculators PPC 3.1, there are other predictors also available, such as S2Dv2, or PEP-FOLD 3.5. To determine if the peptide is disordered IUPred3 or PrDOS can be used (Table 1). Additionally to these, docking (Rathnayake et al., 2017), and quantitative structure-activity relationship (QSAR) (Dowaidar et al., 2017a), have been used for CPP and CPP-cargo assessment.

The association of CPPs to cargoes affects the rate and efficacy of CPP internalization (Ruseska and Zimmer, 2020). For further analysis modeling tools could be used. For prediction, validation and visualization of 3D structures many tools from protein field can be applied. To name a few, Maestro 3D structure modelling software, I-TASSER. For example, new CPPs were derived from the SARS-CoV-2 amino acid sequences using I-TASSER service and characterized with molecular dynamics (MD) simulations (Henao et al., 2022). In case of calculations and simulations it should be kept in mind that these highlight the most probable projections, and should be accompanied by experimental data. Molecular dynamic (MD) simulations provide an approach to inspect specific aspects of the CPPs and their internalization, although at current state, again, they should mainly be used to complement experiments and validate results (Reid et al., 2019). Molecular dynamic simulations were used to characterize amyloidenic peptide-CPP fusion peptide (Likhachev et al., 2022), the absorption and interactions of CPPs on model membranes (Chiarpotti et al., 2021; Mucha et al., 2022), or to model and characterize peptide-siRNA complexes (Purijjala et al., 2022). MD simulations for 24 AI-generated peptides yielded in a Pep-MD peptide with better permeability and weaker toxicity in comparison to Tat, “providing mechanistic information supplementing statistical inference” (Tran et al., 2021). MD simulations have been used to describe the entry of arginine-rich CPPs (Allolio et al., 2018), AMPs (Ma et al., 2020), and Spontaneous Membrane Translocating Peptides (Cao et al., 2020).

##### Modification of CPPs

Although calculations and *in silico* approaches offer several shortcuts for finding probable CPP candidates for cargo delivery, generally several analogs are designed, synthesized or expressed and experimentally tested (Hoffmann et al., 2018; Porosk et al., 2021). As mentioned above, by including modifications new CPPs suiting specific conditions can be generated. It is possible to add a variety of modifications into the peptide sequence to suit specific cargo and applications. For example non-proteogenic amino acid residues or residues associated with specific characteristics, such as the pH-sensitivity of histidine (Porosk et al., 2019), or increase of hydrophobic interactions over tryptophan (McErlean et al., 2021), or design the sequence to self-assemble at certain conditions (Weinberger et al., 2017). More advanced modifications, such as cancer-targeting elements (Feni et al., 2019; Säälik et al., 2019) have been used. If possible, the predictions and calculations should be repeated with the modified version of the CPP or CPP-cargo.

#### Cell-free physico-chemical characterization of CPP-cargo and CPPs

The optimal association between the CPP and cargo depends on the type of CPP, type of cargo, but also the target site, used cells type and experimental conditions. The strategy used for associating the CPP and cargo may affect the efficacy, the internalization route, or even the stability and bioavailability of both. There is a variety of approaches to evaluate the association or stability of the CPP-cargo and physico-chemical properties of the CPP-cargo.

##### Cargos and CPP-cargo association strategies

There are two main strategies to form associations between the cargo and the CPP: covalent linking and non-covalent association. Over covalent conjugation cargoes such as peptides, fluorophores, small chemotherapeutic drugs, peptides, nucleic acids have been attached to the CPPs (Borrelli et al., 2018). There are several linking chemistries available depending on the cargo and application (Tai, 2019). The non-covalent cargo association has been used mainly with nucleic acid cargo, but also for proteins, viral particles (Váňová et al., 2019), and liposomes. The CPP-cargo associations are formed due to weaker forces, such as interactions between negatively charged phosphate backbone of the nucleic acid and the positively charged groups in the amino acid residues of cationic CPPs, also hydrophobic interactions (McClorey and Banerjee, 2018). Although both strategies have their advantages and limits, such as heterogeneity-homogeneity, higher-lower stability, *etc.* the characterization of the CPP-cargo association, dissociation and interactions may help to design more efficient CPP-cargo associations for specific applications. It is important to keep in mind, what is the aim and what would be the end goal for the CPP-mediated delivery. For example, if the aim is to show CPP-cargo dissociation at tumor microenvironment, then the linker between the cargo and the CPP could be pH-sensitive or cleaved with enzymes abundant at that site. Especially if the cargo must be activated by removing the CPP only after reaching the tumor.

There are certain limits associated with some CPPs. For example, if the CPP includes non-proteogenic amino acids or additional modifications, then these peptides must be synthesized. For protein-derived peptides such as tat, there are options of expressing the CPP with the cargo, which can be either protein or peptide. For non-covalent association, the addition of hydrophobic fatty acid, such as in further modifications of transportan10, may offer additional stabilization of the formed nanoparticle. Depending on the cargo and association, slight considerations and modifications to the protocols are required. For example, if nucleic acid is used as the cargo, several intercalating dyes are available. For protein cargo, model green fluorescent protein or other easily detectable proteins are used. The characterization of the CPP-cargo depends also on the limitations the cargo faces. The limitations of using, for example RNA-based therapeutics, include their low stability, negative charge, immunogenicity of exogenous RNA, *etc.* Therefore, the CPP has to offer properties which help to overcome these, and approaches investigating the complex formation, their stability and release are used to elucidate these aspects. Although several modifications can be introduced to the cargo (Amato et al., 2020), the development of the CPP and cargo should be coherent to achieve higher efficacies.

##### Association and dissociation of CPP-cargo

The non-covalent association between the CPP and the cargo is led by weaker forces, and generally an excess of the CPP is required to form stable nanoparticles. For non-covalently associated cargo often gel retardation assay/gel shift assay is used to determine the ratios at which CPP should be associated with the cargo. For example, when using cationic CPP and nucleic acids the negative charges from the NA are neutralized by the positive charges from the CPP the band of the detected NA remains in the gel tooth. Although it does not fully reflect the formation of stable nanoparticles, it gives an indication of required ratios between the NA and CPP. By pre-incubating the CPP-cargo with heparin, FBS, or dissociating the complexes after incubation to determine intact NA, the gel shift assay provides a versatile approach for CPP-cargo characterization. For example both complexation and de-complexation of mRNA and CPP was investigated with gel shift assay to determine complexation ability, and heparin displacement and RNAse treatment to determine the stability of CPP-cargo complexes (Kim et al., 2022a; Kim et al., 2022b).

Similarly to gel shift assays fluorescent dye, such as ethidium bromide, intercalation assays provide a versatile tool for CPP-cargo characterization in solutions and buffers. Additives such as heparin sodium salt, and other compounds that do not affect the binding of the dye, can be used. Additionally, the measurement can easily be done during the incubation time reflecting the dynamics of association, dissociation, and stability to both enzymatic digestion and heparin displacement (Porosk et al., 2019). If the cargo is fluorescently labelled or the cargo is a fluorescent label, then it should be considered when interpreting the results that in case on non-covalent association the signal may be quenched due to packing, and the readout is rather qualitative not quantitative.

In case of hydrophobic CPP-cargo the potential of these to self-assemble into nanostructures can be investigated by detecting an abrupt change in absorption associated with micelle formation. The concentration at which this occurs is defined as critical micelle concentrations (CMC). The CMC has been used to characterize CPPs (Eiríksdóttir et al., 2010) and CPP-cargo conjugates (Esbjörner et al., 2007; Lützenburg et al., 2021), and self-assembly of oligoproline peptide (Kawasaki et al., 2020).

Nuclear magnetic resonance spectroscopy (NMR) is used to investigate the physical, chemical and biological properties of a matter, for example the position of CPP penetratin in SDS micelles, its conformation in membranes, or CPPs in model membrane systems (Lindberg et al., 2001; Su et al., 2008; Mäler, 2013). More recently NMR was used for characterization of MeCP2-CPP peptides (Beribisky et al., 2022).

Mass spectrometry (MA) is an analytical technique that separates ionized particles by using differences in the ratios of their charges to their respective masses (mass/charge; m/z) and can be used to determine the molecular weight of the particles. Matrix-assisted laser desorption/ionization–tandem time-of-flight (MALDI-TOF) mass spectrometry is often used to confirm the molecular mass *versus* charge (m/z) of the peptides. MALDI-TOF mass spectrometry has also been used to quantify cellular uptake of CPPs (Burlina et al., 2005; Aubry et al., 2010; Bechtella et al., 2022). By combining a proteomics approach by combining MS and sample enrichment, the interacting partners, actin and albumin, for CPPs (R/W)_9_ and (R/W)_16_ (Clavier et al., 2014), or the stability of the CPP-PMO (Schissel et al., 2022) have been characterized. Mass spectrometry is a direct quantification tool that would give information about the concentration of peptides recovered from biological mixtures with limited labeling required.

Liquid chromatography (LC) methods can be used for assessment of both the CPP and CPP-cargo. LC-MS joins both liquid chromatography and mass spectrometry. Chromatography-based approaches are also often used for characterization of the peptide or CPP-cargo. More advanced chromatography methods have also been developed. For example, immobilized artificial membrane chromatography was used to characterize CPP TP10 and other AMPs (Ciura et al., 2021). HPLC is often used for purification of the CPP-s post-synthesis, but it can also be used to characterize CPP-cargo, and UPLC methods can be used to characterize the stability of CPP-cargo by detecting its degradation. Spectroscopy-based methods such as Fourier Transform infrared spectroscopy (FTIR) was used to characterize R8-cargo complexes (Ratrey et al., 2022), and the presence of CPPs in the gold NPs was confirmed by this method (Boussoufi et al., 2018).

##### Size, size distribution and surface charge of CPP-cargo

Size and particle size distribution are one of the important features of CPP-cargo associations. The conventional size of non-covalent CPP-cargo associations range around 100 nm, and commonly compose of different size complexes. Especially in the case of non-covalent association the size of the complex at different CPP to cargo mixing rations help to decide the optimal conditions for nanoparticle formation. For this, measurements with dynamic light scattering (DLS) are often used. The application of DLS techniques for nanoparticle characterization and development are briefly discussed in a review (Carvalho et al., 2018). As an example of DLS-based characterization, the formation between CPPs and nucleic acid cargo was extensively assessed, revealing the differences between complexes PepFect14/NA and PepFect6/NA (Margus et al., 2016). DLS is also useful when determining optimal buffers for CPP-cargo formulations (Beribisky et al., 2022). For characterization of CPP-cargo morphology, size, and size distribution electron microscopy (EM) based approaches have been used. For example, the size, morphology and distribution of size-range of histidine-containing peptides non-covalently associated with siRNA (Porosk et al., 2019) has been characterized. The main disadvantage of these kind of methods are that they are operated under high-vacuum which may affect the samples, nevertheless, this type of characterization is often included, especially if the CPP-cargo associations are considered novel.

Charged CPP-cargo assemblies in solutions attract counter ions to their surfaces forming a layer around the particle. The ζ-potential (zeta-potential), which can be calculated from electrophoretic mobility of the CPP-cargo associates when an electric field is applied, and the intensity fluctuations of scattered light from the particle, correlates with the surface charge of the particle at given conditions (Padari et al., 2019). The measurement of zeta-potential enables to determine the ratios at which the surface charge of the CPP-cargo changes, indicating an optimal range where nanoparticles are formed (Kurrikoff et al., 2017; Padari et al., 2019). For nucleic acid cargo, the negative charges can be neutralized by the CPP, and by additional modifications, such as addition of PEG may result in non-covalent associations with low zeta-potential. This reduces the possibility of the CPP-cargo to interact with serum proteins, or reduce the clearance. Additionally, other ensemble methods such as small-angle X-ray scattering (SAXS) can be used. SAXS is a suitable method which can help to characterize the size, shape and packing of CPP-cargo, for example CPP/DNA (da Silva et al., 2022).

##### Secondary structure of the CPP and CPP-cargo

In the design and modification of the CPPs and CPP-cargo not only the peptide sequence, but additional features such as secondary structure or flexibility affect the delivery efficacy of the CPP (Kalafatovic and Giralt, 2017). The alpha-helical structure has been shown to be beneficial for internalization, and for rational design of their peptides several groups have used helical wheel predictors as basis of design (Freimann et al., 2016). For this purpose, for example Heliquest (Gautier et al., 2008) among others, can be used. Although this projection can accompany the measurements from circular dichroism (CD) spectra, the CD spectra does not determine the exact location of the alpha helix in the peptide, therefore it has a supportive role rather than concrete interpretation of secondary structures. CD is a method based on differences of absorption of left- and right-handed circularly polarized light by chiral molecules. In current context it is useful for determination of the secondary structure of peptides in different conditions. These may include the presence of buffers and/or micelles/vesicles mimicking conditions of cell membranes or near cells (Jobin et al., 2019), or in the presence of cargo, showing the changes in peptide when interacting with nucleic acid (Vasconcelos et al., 2014). As the secondary structure of the CPP may be of importance, CD also allows to monitor the effect of introduced amino acid changes to the peptide. For example, the increase or stabilization of helical structure in the peptide (Freimann et al., 2016), or the effect of introduction of Aib into the sequence and its effect on the uptake (Takada et al., 2022). The secondary structure calculations from the CD measurements requires software accompanying the machine, or online tools such as BeStSel (Micsonai et al., 2022). There are also several servers for prediction of secondary structures (Table 1).

For covalently linked CPP-cargo, there may be interactions between both, which may lead to decrease of cell-penetration or of the bio-effect the cargo should have. To overcome this, strategies, such as cleavable linked between the CPP and cargo, or shielding with PEG have been used. Especially in case of covalently linked CPP-cargo, the linking strategy must be chosen so both retain their efficacies.

#### Internalization of CPP-cargo into the cells and their trafficking in the cells

Beyond CPP-cargo association, the next steps for CPP-cargo are interactions with extracellular components and cell membranes, including interactions with receptors, and internalization following intracellular trafficking.

##### Interactions with (cell and artificial) membranes

Often the secondary structure goes together with membrane insertion potential of the CPP and CPP-cargo. Interactions with the cell membrane are essential for the peptide and CPP-cargo to internalize into the cell and is one of the first steps required for the internalization. To some degree these interactions can be predicted, with predictors such as CELLPM 2.0 (Lomize et al., 2021). It has been suggested that the destabilization of the cell membrane can be initiated by the accumulation of positively charged CPPs on a negatively charged membrane surface by attracting water molecules that bind to the charged amino acids of CPPs into the hydrophobic core of lipid bilayers (Grasso et al., 2018), or by inducing transfer pores in the membrane (Islam et al., 2018).

Experimentally the membrane activity of the CPPs and CPP-cargo can be evaluated on red blood cells (RBC). In current context the RBC are regarded as simplified models of cells. RBCs have been used for assessing *ex vivo* the membrane activity and the endosomolytic properties of CPPs if corresponding conditions are used (Porosk et al., 2019). The idea is that when the cell membrane is compromised, the heme is released from the cell and the increase of absorption is relative to the membrane activity. Although hemolysis is often used also as a toxicity assay, it should be noted that the membrane activity of CPPs drastically decreases if serum proteins are present in the cell suspension. Therefore, in order to measure membrane activity, all traces of these proteins should be removed by several washing steps. The hemolytic activity has been regarded as both reflection of membrane activity and potential toxicity. To calculate hemolytic properties the CPPs predictor such as HemoPI (Chaudhary et al., 2016; Vita et al., 2019) can be used.

In addition to RBC, the interactions or leakiness can be investigated on more artificial setups, such as large unilamellar vesicles (LUV) and giant unilamellar vesicles (GUV) (Ratrey et al., 2022). By enclosing fluorescent dyes into the vesicles, the leakage of these detectable substances can be measured after interactions between the CPP-cargo and vesicles. The composition of the vesicles can be varied to model different membranes. As an example, the effect of membrane potential to the entry of TP10-cargo was investigated on GUVs (Moghal et al., 2020).

##### Internalization and cellular localization

The CPPs and CPP-cargo associations employ a great diversity of routes to enter the cells. There are indications for CPPs entering *via* direct penetration/translocation, but often, especially when associated with cargo, the endocytosis pathways are harnessed (Xu et al., 2019; Yang et al., 2019; Sakamoto et al., 2020). By endocytosis the CPP-cargo may remain in endosomes and be routed for degradation to lysosomes. Endosomal entrapment and following endosomal escape are required for efficient delivery (Borrelli et al., 2018; Xu et al., 2019). There have been also indications of alternate routes for CPP endocytosis *via* a newly discovered Rab14-dependent but Rab5-and Rab7-independent pathway, and interactions with potassium channels in case of TAT-cargo direct translocation (Trofimenko et al., 2021). Most of the studies involving assessment of CPP-cargo efficacy and investigations of their internalization and delivery mechanisms are carried out on cell cultures. There are also predictors for cellular localization, such as DeepTMHMM (Hallgren et al., 2022).

The CPP-cargo internalized by endocytosis pathways often ends up in the late endosomes and lysosomes, where degradation takes place. To have its intended effect in the cell, the cargo must escape the endosome before that. The endosomal escape is one of the crucial bottlenecks not only for CPP-mediated delivery but generally for non-viral delivery vectors. There are several mechanisms of escaping the endosome mediated by the CPPs. One of these is proton sponge effect, where endosomal swelling causes endosome to rupture and release its contents. Increasing evidence suggest the presence of other escape mechanisms. There are many strategies to overcome endosomal entrapment and enhance the endosomal escape (Pei and Buyanova, 2019). Depending on the cargo, the target may locate in the cytosol of the cells, for example, for RNA-based cargo, or if the cargo is for example plasmid DNA, then the cargo has to reach the nucleus. Therefore crossing of two membrane barriers is required. After endosomal escape or during endosomal trafficking the cargo, especially non-covalently attached cargo, must be released from the nanoparticles for the bio effect, such as gene downregulation, to realize. Therefore evaluation not only the end-point of achieving biological effect from the cargo, but also the mechanisms behind the delivery must be done. Because both internalization, trafficking and cargo reaching the destination are crucial bottlenecks for efficient delivery, several methods are used to investigate these.

To experimentally evaluate the internalization and trafficking of the CPP-cargo, there are some common methods that are used and varied. Often flow cytometry and microscopy based approaches are applied (Szabó et al., 2022; Zorko and Langel, 2022) accompanied with fluorescence detection. For example, the assessment of the CPP backbone rigidity effect (Nagel et al., 2017), or a standardized evaluation of 474 sequence motifs by Remaker et al. (Ramaker et al., 2018). The way the cells are treated, fixed, or prepared for the analysis, may affect the output, leading to a high variety in the results or even contradictions although the same CPP is used. The widely used fixation methods may affect the result (Richard et al., 2003), therefore, if possible, the live cell imaging and analysis are preferred. The delivery capacity of five CPP sequences – penetratin, R8, Tat, Transportan and Xentry (Patel et al., 2019), or the application potential for targeted sub-cellular delivery with cyclic CPP (Schneider et al., 2021) have been investigated on live cells. More advanced approaches, such as single particle tracking allows to observe the interactions of individual nanoparticles, their interactions, trafficking and how it affects the infrastructure of the cell during trafficking. With this CPP-nanoparticles (Streck et al., 2021), R8-Pdots (Luo et al., 2019) have been investigated. Additionally, artificial vesicles can be used instead of cells (Ciobanasu et al., 2010). The total quantitation of reporter product has been often used as a measurement of general efficacy, as it does not only reflect the internalization, but also the uptake, trafficking, endosomal escape, and release of the cargo and summaries the final cargo that has been successfully reached its target and is bioactive. Additionally to detection of fluorophores or fluorescent proteins post-transfection, luminescence-based approaches have been widely used. The luciferase activity form delivered protein, expression vector or even RNA is offers sensitive, quantifiable and broadly used approach (Ruseska and Zimmer, 2020).

As an alternative to fluorescent labels and luminescence base molecules, other methods for cargo or peptide quantitation with limited or even without the need of labelling would be beneficial for understanding the internalization and trafficking. Especially as certain modifications, linkers and fluorescent tags may affect the CPP-cargo properties. For example, the quantitation of antisense oligonucleotide analog without the need for labeling, nevertheless it requires subcellular fractionation after internalization, RNA preparation and enzyme-linked immunosorbent assay (Pendergraff et al., 2020). In another work KITENIN was detected from cytosol and membrane fraction (Kim et al., 2022a). Other approaches, such as radiolabeling (Abbasi Gharibkandi et al., 2020), subcellular fractionation from the cytosol and whole cell lysate accompanied with MALDI-TOF based analysis have been used (Schissel et al., 2022).

##### Uptake pathway

Uptake pathway can be revealed by using EM-approaches and CPP-cargo co-localization with certain organelles. Often inhibitors are used in cell experiments described above to evaluate the role of a certain internalization pathway in the uptake of the CPP-cargo. Although their use pose several limitations. For instance, most of inhibitors are chemical compounds that may affect other processes or even the CPP-cargo itself, therefore their specificity is sometimes limited. Interestingly, when limiting internalization *via* one route, the CPP-cargo can be taken up more efficiently by other. The optimal concentrations vary between cell lines, experimental conditions, therefore correct controls should be included. Additionally, it is often impossible to completely block the given pathway, and references and controls should be included where possible (Vercauteren et al., 2010; Georgieva et al., 2011; Dutta and Donaldson, 2012). For example, the compounds such as chloropromazine hydrochloride, nocodazole, geinstein, methyl-beta-cyclodextrin, chloroquine, filipin, are only a few that have been used. In addition to using chemical compounds, downregulation of specific receptors, weather transiently or in stable modified cell lines, has been used to reveal possible partners for CPP-cargo. Other approaches, such as transferring the cells to 4°C to limit the use of endocytosis pathways or ATP depletion have been used. Again, the readout is often based on fluorescent detection methods, or changes in the reporter levels detected otherwise.

##### Internalization into advanced cellular models

To positively identify the most effective CPPs for clinical trials, it is recommended to use cellular 3D models, as these are a step closer to *in vivo* conditions when compared to traditional monolayer cell cultures (Reissmann and Filatova, 2021; Cacciamali et al., 2022), especially in case of pathological tissues. Compared to monolayer cultures, the 3D cultured cells have been shown to have an increased resistance to therapeutics (Jensen and Teng, 2020). These can include more than one cell type, and create more *in vivo*-relevant models. For example penetration of cationic CPPs R9 and penetratin, or therapeutic potential of PepFect14/mRNA nanoparticles in 3D ovarian cancer spheroids and patient-derived 3D tumor explants have been investigated (van den Brand et al., 2018; van den Brand et al., 2019). In tumor and endometriotic 3D models the downregulation of target genes with PepFect6/siRNA and NickFect70/siRNA was investigated (Kiisholts et al., 2021). In colon cancer 3D models, the efficacy of CPP was assessed (Al-HusainiElkamel et al., 2020).

### Functional evaluation

The functional evaluation of the CPP-cargo includes the evaluation of the biocompatibility/toxicity, biodistribution *in vivo* and evaluation of CPP-cargo treatment affecting cell functions. Although the reporter assays provide excellent primary indications of possible applications, the efficacy of the CPP-cargo for specific applications must be tested with relevant cargos and at relevant conditions. For *in vivo* evaluation there are several aspects, such as administration route.

### Toxicity and biocompatibility of CPP and CPP-cargo *in vitro* and *in vivo*

**FIGURE 3:**
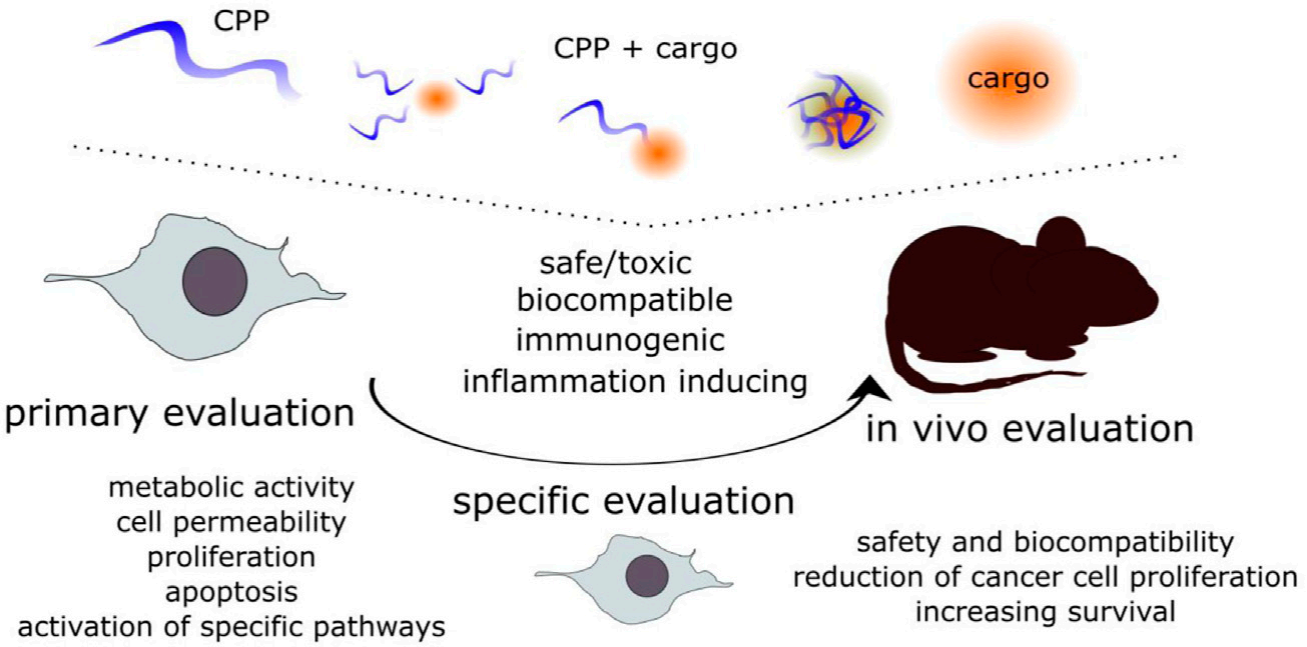
The evaluation of the safety, biocompatibility and bioeffect of the CPP-cargo. The CPP, the cargo and the CPP-cargo associations should be evaluated at each step. The initial primary evaluation is done with more general approaches, and after confirming the safe or effective doses more specific approaches can be used to determine effects on different aspects, such as reduction of cell proliferation, increase of membrane permeability, cell cycle arrest, *etc.* These are also required for further evaluation *in vivo* to confirm safety of the CPP-cargo or if the CPP-cargo is able to reduce the proliferation of cancer cells and increase survival.

Following thorough physiochemical assessment and confirmation of cell entry or bio effect, the toxicity and biocompatibility should be tested *in vitro*. For further *in vivo* experiments, the *in vitro* assessment is a must. Natural processes such as cell growth, division, repair, and apoptosis can be affected by substances in the extracellular environment and in the cells. Biocompatibility and maintenance of viability of normal cells are another aspect to consider in CPP-mediated delivery. On the other hand, if the target of the CPP-cargo is to reduce the proliferation of aberrant cells, such as cancer cells, the reduction of viability for those cells would be welcome (Zhou et al., 2022). Although some general online tools (Table 1) are available for predicting toxicity, or immunogenicity, allergenicity, the possible effects should be confirmed experimentally. We have combined the assays used both *in vitro* and *in vivo* under one section. The initial screening in cultured cells (primary evaluation), is followed by more specific evaluations and for preclinical assessment, also in *in vivo* models (Figure 3).

Cytotoxicity assays reflect the effect CPP or CPP-cargo can have on the cell, leading to cell progression into death phase, activation of controlled cell death, cell lysis or activation of autophagy. *In vitro* cytotoxicity assay methods can be broadly divided into assessment of proliferation, metabolic state, cell division, changes in morphology, or membrane integrity. Nevertheless, no *in vitro* assay can fully reproduce the intricate and complex interactions in the animal or human, and for clinical applications *in vivo* safety and bio effect should be confirmed. Additionally, as opsonization of nanoparticles has been suggested, evaluation of CPP-cargo after interacting with host serum may give insight of possible limitations and hints for improvement.

CPPs and CPP-cargo are often assessed by using assays reflecting the metabolic state of the cells. For this MTS/MTT/XTT/WST-1 based tetrazolium reduction assays, quantification of intracellular ATP, glucose consumption can be used. In more thorough analysis by metabolic profiling on CPP-treated cells it was revealed that CPP induced alterations of intracellular redox potential (Kilk et al., 2009). Similarly to other assays, the conclusions of CPP-cargo safety and effect on cells should be drawn on the basis of more than one assay. An excellent example of this is the characterization of R7-G-Aβ_25-35_ peptide (Mendivil-Perez et al., 2022) with more general assays such as MTT, and then to reveal more specific aspects other flow cytometry based assays were introduced. The toxicity and immunogenicity of PepFects was assessed with assays reflecting both viability and immunogenicity of the CPP-cargo on cells in culture and *in vivo* (Suhorutsenko et al., 2011). Regardless of their broad use, the metabolic assays generally do not represent the changes in cell growth or other effects on the cells. The cell cycle consists of several phases and check points, which can be reflected with specific assays. BrdU-based assay, which reflects the integration of the nucleoside analog into the new strands of DNA synthesized during DNA replication, has been used to assess the effect of CPPs and other transfection reagents (Porosk et al., 2022). Ki-67 has been used as a proliferation marker for human tumor, although it reflects other additional processes (Sun and Kaufman, 2018). Apoptotic cells can be detected by flow cytometry accompanied by specific staining, with TUNEL assay (DNA fragmentation) or Annexin V-FITC/PI double staining assay. Live/dead assay, which reflects both the viable cell population and cells with damaged membranes, is compatible with both flow cytometry and microscopy (Porosk et al., 2022). Caspase 3 assay is also useful when assessing pro-apoptotic effect of anticancer treatment.

Autophagy is a regulating cell mechanism which helps the cell to remove and recycle nonfunctional components. The induction of autophagy is broadly evaluated by measuring directly the levels of lactate dehydrogenase (LDH) and other proteins. Other approaches, for example, RNA sequencing was used to characterize the cells transfected with CPP-ASO, and revealing the induction of autophagy post-transfection (Dowaidar et al., 2017b). As an example, the induction of autophagy and apoptosis after treatment with CPP TAT modified DNA tetrahedrons was assessed in colorectal cancer cells and additionally the *in vivo* safety of these constructs (Zhang et al., 2019). If the CPP-cargo should help to reduce the effect of some cytotoxic compounds, then often the cells are pre- or co-treated with the compound followed by cytotoxicity assay. For example for assessing neuroprotective effect mediated by CPP-cargo, MTS assay accompanied with MPP+ pre-treatment helped to investigate this (Shalaby et al., 2022).

Preferably the CPP and CPP-cargo should be not only biocompatible, but also immunologically inert. Different immune-assays can be used, such as determination of IL-1β, IL-18, and TNF-α cytokine release (Suhorutsenko et al., 2011) post-treatment, which reflect the activation of the immune system. This can be the result of introduction of foreign material, such as proteins, peptides, nucleic acids. This kind of activation may compromise the clinical effect (Suhorutsenko et al., 2011) and should be avoided. In contrast, for antigen vaccine applications the CPP should promote the endocytosis, uptake of the CPP-cargo into the cytosol of the antigen presenting cells leading to an increase of its potency. CPP-peptide immunization has been tested and it promoted antigen trafficking to lymph nodes, improved antigen stability and prolonged robust antigen presentation compared to free peptide antigens (Backlund et al., 2022). CPP-based strategies for immunomodulation have also been suggested recently (Koo et al., 2022). The evaluation of inflammation responses such as upregulation of nuclear factor kappa β, activator protein, extracellular signal regulated kinases c-Jun, N-terminal kinases, have also been used to characterize CPP-cargo *in vitro* and *in vivo*. Effect of anti-inflammatory peptide with CPP properties AIP6 (Wang et al., 2011) was confirmed by reduction of inflammation induced by subdermal injections of Zymosan. The level of cytokines in the serum can be detected from blood samples of treated mice post-injection (Suhorutsenko et al., 2011).

Often histological examination of *in vivo* samples may reveal additional concerns regarding the CPP-cargo, and delivery efficacy. From the histological (histo-pathological) examination differences between the treated and untreated should be considered. Histological examination of the major organs has been used to confirm the safety of the CPP-cargo (Zhang et al., 2019). Additionally, detection of the levels of specific genes, such as TNF-α, IL-1β, IL-6, inducible nitric oxide synthase, and cyclooxygenase-2 mRNA has been used (Wang et al., 2011). In case of specific treatments, levels of affected expression may be also quantified. For example, the blood coagulation factor VII expression after administration of CPP-siRNA nanoparticles (Porosk et al., 2019).

### Biodistribution

As and additional level, not only the processes taking place in the delivery into cells in cell cultures, but also pre-clinical investigations of CPP-cargo efficacies in much more complex models *in vivo* are required. *In vivo* delivery poses several more aspects, such as biodistribution, serum stability, opsonization, or premature cargo release, which *in vitro* testing can not model. To improve targeting phage display methods have been successfully used to identify tumor homing peptides addressing specifically blood/lymphatic vessels of tumors as well as to various normal tissues, which have been used as tissue-specific biomarkers of the normal and diseased vasculature yielding targeted therapeutics and imaging agents to tumors. *In vivo* phage display technology, based on phage libraries, in which each individual phage expresses a unique peptide sequence or protein fragment on its surface revealed multiple tumor homing CPPs (Eriste et al., 2013; Martins et al., 2016; Põšnograjeva et al., 2022).

The challenges the CPP-cargo must overcome depend highly on the chosen administration route. Local injection into a tumor or specific organ, intravenous administration, intraperitoneal and intramuscular injection, are the most used routes for CPP-cargo administration. Recently, intranasal administration has also surfaced as one of the promising routes (Akita et al., 2021). Each of these pose different challenges and advantages over each other. The systemic delivery enables the CPP-cargo to reach different organs but requires improvement of targeting. Local injection into tumor is suitable for proof-of-concept but does not reflect the administration route which could be useful for therapeutic downstream therapeutic purposes. Intranasal administration is less invasive and enables delivery into the brain. The *in vivo* assessment can be broadly divided into effects measured *in vivo* and *ex vivo* after harvesting the organs or tissues. In case of *in vivo* assessment imaging is often used, although functional assays such as reduction of tumor growth post-treatment can be used. *Ex vivo* assessment improves the accuracy of the generalizations as organs or tissues are separated from and the changes evaluated between the groups. From *in vivo* experiments one would like to verify the internalization, biodistribution, safety, and finally bio-effect of the CPP-cargo. The biodistribution of the CPP-cargo is one of the first indications of the organs and tissues the particle reaches. Biodistribution is often assessed by adding a fluorophore, quantitation of expression levels, total reporter protein levels, such as luciferase from whole tissue homogenate (Freimann et al., 2016). Nevertheless, biodistribution may not reflect the bioeffect the CPP-cargo may have, as bioeffect is not always correlating with accumulation of the particles or labels in certain tissues. Colocalization of specific cell types or effects of the treatment on the tissues can be assessed by immunohistochemical treatment of the extracted tissues followed by analysis. For example, the presence of inflammation in lungs post treatment with CPP-cargo in mice with LPS-induced acute inflammation assessed on lung tissues staining with hematoxylin and eosin (Kurrikoff et al., 2019). *In vivo* assays also help to understand the pharmacokinetics, more precisely the absorption, distribution, metabolism, and excretion, of the CPP-cargo. Ten CPPs have been evaluated based on the binding to specific cell lines, biodistribution *in vivo* and biodistribution in tumor bearing mice (Sarko et al., 2010). In tumor bearing mice the labelled CPPs showed rapid blood clearance, and accumulation in liver and kidneys already at 10 min post-injection. After 4 h most of the CPPs were retained in the liver and kidneys (Sarko et al., 2010). In the same work, the imaging of CPP-treated rats revealed broader localization of transportan compared to penetratin and tat (Sarko et al., 2010). As a more recent example, the TAT-antibody conjugate mean tissue concentrations in tumor-xerograph mouse model were investigated (Polli and Balthasar, 2022). In case of CPP-plasmid non-covalently formed nanoparticles, after systemic administration *via* tail vein, the expression from delivered plasmid was detected in the lungs, liver, and spleen 24 h post-injcetion (Freimann et al., 2016). To improve the delivery, the CPP-cargo ratios may require further optimization. For example, reduction of plasmid dose and CPP to nucleic acid ratio (Kurrikoff et al., 2017).

### Evaluation of bio-effect

In cancer cells there are specific traits, such as cell proliferation, angiogenesis, metastasis, immunosuppression and resistance to chemotherapy which can be addressed to improve treatment. These aspects can be evaluated using approaches described above. Such as cell cycle arrest after treatment, reduction of cancer cell viability, but also improved effect after CPP-cargo and drug co-treatment. For example, the chitosan lactate nanoparticles functionalized with TAT and hyaluronate to deliver doxorubicin and siRNA against CD73 suppressing the angiogenesis, invasion, proliferation and migration of cancer cells (Salehi Khesht et al., 2021). Tumor tissue invasiveness and migration on 3D models has also been assessed (Kiisholts et al., 2021). These can be evaluated on 2D cell cultures, but also by using transwell-based models, spheroid-based models, hybrid models or tumor-microvessel models (Katt et al., 2016). The *in vitro* models allow to investigate aspects of the CPP-cargo response to modeled tumor microenvironment in a controlled manner. Nevertheless, the choice of model depends on the application of the CPP-cargo.

There have been already a set of murine models for preclinical *in vivo* evaluation of potential cancer therapeutics (Olson et al., 2018; Guerin et al., 2020). The tumors include spontaneous and transplanted tumors. The tumor characteristics depend on the origin and the cell type, location, and the mouse strain. The tumors may orignin form established cell lines, primary tumor cells from spontaneous tumors, or fragments of tumors transferred from donor to host animals (Guerin et al., 2020). In case of murine tumor models, the reduction of tumor growth, increase of survival have often been used as evaluation approaches. The tumors can consist of specific cell types, to model different tumors with varying degrees of invasiveness, growth, vascularization and metastasis. For example the reduction of tumor size after treatment with STRAP-4-MTX was assessed both by imaging and comparison of excised tumor and spleen (Jerath et al., 2022). PEGylated docetaxel nanocrystals modified with TAT enhanced cellular drug uptake, leading to stronger cell growth inhibition of TC-1 model tumors in mice (Lv et al., 2021).

In case of neurodegenerative diseases CPP-cargo have also seen as a promising approach (Xie et al., 2020). In neurodegenerative diseases there have been several murine models for different neurodegenerative diseases, such as Parkinson’s disease or frontotemporal dementia, and aspect, such as progressive loss of dopamine neurons, loss of upper and lower motor neurons, or cognitive impairment, related to these (Dawson et al., 2018). The evaluation post-treatment with CPP-cargo is based on the improvement or reduction of decline.

Additionally to cancer and neurodegenerative disease treatment applications, the CPPs could be used as delivery vectors for alleviating inflammation (Wang et al., 2011; Kurrikoff et al., 2019), including asthma (Kurrikoff et al., 2019) or contact dermatitis (Carreras-Badosa et al., 2020), blood coagulation disorders (Porosk et al., 2019), and endometriosis (Kiisholts et al., 2021). There have been several CPP-related compounds in preclinical and clinical trials for cancer treatment, such as PEP-010 for metastatic solid tumor treatment or AVB-620 for breast cancer treatment (Matijass and Neundorf, 2021). Or CPP-cargo for central nervous system disorders, such as K16ApoE for Alzheimer’s disease or TP10-dopamine fusion protein against Parkinson’s disease (Xie et al., 2020). Recently the first cell-penetrating peptide technology based treatment DAXXIFY^™^ was approved. In order to predict *in vivo* efficacies from based on *in vivo* data, the set of experiments have to be chosen mindfully (Bouhaddou et al., 2020). Nevertheless, at this point, not every interactions and mechanism is known for every disease, its progression and treatment targets. With over 80 peptide drugs approved worldwide (Wang et al., 2022), using peptides as therapeutics or carriers has become more and more appealing.

## Conclusion

To adequately assess the efficacy of CPP-cargo system many aspects have to be considered. These include the association of CPP-cargo, detailed records and relevant characterization of the CPP-cargo and using a relevant set of approaches for the characterization. With increasing merging of CPPs into other delivery methods, such as LNPs, evaluation approaches can be adapted from other fields as well. Today there are increasing number of servers for prediction and characterization of both peptides and cargoes, which can help to further optimize, design, and characterize CPP and CPP-cargo without the need to experimentally test each of them. A large proportion of work has been done to characterize the CPP-cargo, their internalization and trafficking. Nevertheless, for more relevant characterization, the set of approaches has to be chosen mindfully to reflect the aspects crucial for the end-point of the CPP-cargo, for example, cancer therapy. Although there are cell free and *in vitro* models for initial characterization, *in vivo* experiments are still a major part of the evaluation, and the *in vivo* models must be considered thoroughly. As an additional challenge, as there are a wide variety of evaluation approaches, the field lacks standardized evaluation approaches. Additionally, with the increasing possibilities for CPP-mediated delivery, the standardization may become increasingly complicated.
